# Fetal size in the second trimester is associated with the duration of pregnancy, small fetuses having longer pregnancies

**DOI:** 10.1186/1471-2393-8-25

**Published:** 2008-07-16

**Authors:** Synnøve L Johnsen, Tom Wilsgaard, Svein Rasmussen, Mark A Hanson, Keith M Godfrey, Torvid Kiserud

**Affiliations:** 1 Department of Obstetrics and Gynaecology, Haukeland University Hospital, Bergen, Norway; 2 Institute of Community Medicine, University of Tromsø, Norway; 3 Medical Birth Registry of Norway, Locus of Registry Based Epidemiology, University of Bergen and the Norwegian Institute of Public Health, Norway; 4 Department of Clinical Medicine, University of Bergen, Norway; 5 Centre for International Health, University of Bergen, Norway; 6 Division of Developmental Origins of Health and Disease, University of Southampton, Southampton, UK

## Abstract

**Background:**

Conventionally, the pregnancy duration is accepted to be 280–282 days. Fetuses determined by ultrasound biometry to be small in early pregnancy, have an increased risk of premature birth. We speculate that the higher rate of preterm delivery in such small fetuses represents a pathological outcome not applicable to physiological pregnancies. Here we test the hypothesis that in low-risk pregnancies fetal growth (expressed by fetal size in the second trimester) is itself a determinant for pregnancy duration with the slower growing fetuses having a longer pregnancy.

**Methods:**

We analysed duration of gestation data for 541 women who had a spontaneous delivery having previously been recruited to a cross-sectional study of 650 low-risk pregnancies. All had a regular menses and a known date of their last menstrual period (LMP). Subjects were examined using ultrasound to determine fetal head circumference (HC), abdominal circumference (AC) and femur length (FL) at 10–24 weeks of gestation. Length of the pregnancy was calculated from LMP, and birth weights were noted. The effect of fetal size at 10–24 weeks of gestation on pregnancy duration was assessed also when adjusting for the difference between LMP and ultrasound based fetal age.

**Results:**

Small fetuses (z-score -2.5) at second trimester ultrasound scan had lower birth weights (p < 0.0001) and longer duration of pregnancy (p < 0.0001) than large fetuses (z-score +2.5): 289.6 days (95%CI 288.0 to 291.1) vs. 276.1 (95%CI 273.6 to 278.4) for HC, 289.0 days (95%CI 287.4 to 290.6) vs. 276.9 days (95%CI 274.4 to 279.2) for AC and 288.3 vs. 277.9 days (95%CI 275.6 to 280.1) for FL. Controlling for the difference between LMP and ultrasound dating (using HC measurement), the effect of fetal size on pregnancy length was reduced to half but was still present for AC and FL (comparing z-score -2.5 with +2.5, 286.6 vs. 280.2 days, p = 0.004, and 286.0 vs. 280.9, p = 0.008, respectively).

**Conclusion:**

Fetal size in the second trimester is a determinant of birth weight and pregnancy duration, small fetuses having lower birth weights and longer pregnancies (up to 13 days compared with large fetuses). Our results support a concept of individually assigned pregnancy duration according to growth rates rather than imposing a standard of 280–282 days on all pregnancies.

## Background

It was Nägele and his contemporaries who first suggested counting 40 weeks from the first day of the last menstrual period (LMP) to predict the day of confinement [1]. Subsequently, WHO has also defined the normal length of pregnancy to be 40 weeks (280 days)[2], but studies of population-based birth registries suggest a longer pregnancy duration based on LMP (mean 281–283.6 days) [3]. A problem with the LMP-method is that 45–68% of women have irregular periods or uncertain information of their LMP [4,5]. Moreover, the fertile window occurs over a range of days in the menstrual cycle [6]. Ultrasound dating was thought to overcome some of these problems by using fetal size to determine gestational age and thus to predict day of confinement independently of LMP. Based on fetal biparietal diameter (BPD) in the second trimester, pregnancy duration is calculated to be somewhat shorter (mean 280.6 days) [3] than previously thought. Today ultrasound dating has spread to common use and has had the clinically desirable effect of reducing the number of inductions of labour for presumed post-term pregnancies [7].

While ultrasound dating is useful for those women with uncertain LMP, it is less obvious that this is also valid for pregnancies with reliable information of a regular LMP. Even in this group, ultrasound dating does, however, predict day of confinement more precisely than LMP [8-10]. As a consequence ultrasound dating has been recommended as the preferred dating method[7], although this view has repeatedly been disputed [11]. The reason is that charts for ultrasound dating are based on fetal biometry in pregnancies with certain and regular LMP in the first place. It therefore seems unlikely that the ultrasound method could better predict day of confinement than the LMP itself, unless the ultrasound method also includes a factor that is not yet accounted for.

We hypothesize that, in addition to LMP, fetal growth (reflected in fetal size) might be such a determining factor for pregnancy duration. The aim of the present study was therefore to assess the effect of second trimester fetal size on the duration of pregnancy and the influence of ultrasound dating.

## Methods

The present cross-sectional study is part of the larger "Fetal Age and Growth" project that included 650 participants according to a protocol approved by the Regional Committee of Medical Research Ethics (REK-III no. 025.01) [12]. The participants were included after written informed consent provided they were healthy women with no history of complications in previous pregnancies, had exact date of the LMP, a history of regular menstrual periods (28 ± 4 days) for at least three months before pregnancy, and no use of hormonal therapy or contraception in this period. Eight women were excluded because the discrepancy between ultrasound and menstrual age was more than 14 days. In the present study we included only the spontaneous deliveries, which left a study population of 541 women. Gestational age was computed from the LMP and corrected for length of cycle different from 28 days, and participants were examined once between gestational age 10 and 24 weeks, determined according to LMP. Head circumference (HC) was obtained using an ellipse in a horizontal section at the level of the thalamus and the cavum septi pellucidi [13]. Abdominal circumference (AC) was also obtained using an ellipse, in a transverse section of the fetal abdomen at the level where the umbilical vein enters the liver. The femur length (FL) was measured in longitudinal section by placing the callipers at the end of the diaphysis in an image showing both epiphyses [14]. Three measurements were made of each parameter and the mean used in the statistics. Two persons, experienced in performing ultrasound scans performed all the examinations, using Philips HDI 5000, Seattle, or Aloka Prosound-5000, Tokyo.

The length of the pregnancies was calculated from LMP and birth weights were noted.

### Statistical analysis

To achieve normal distribution of pregnancy duration, birth weight, HC, AC, and FL, we used the Box-Cox transformation. Pregnancy duration was raised to the power of 10 but no transformation was found necessary for the other measurements. Fractional polynomials were fitted to find the best relationship between each biometric measurement of fetal size and gestational age [15]. The standard deviation (SD) score (z-score) for each observation was calculated as the distance in SDs from the mean regression line. The method of scaled absolute residuals was used to model SD against gestational age [15]. The association between the transformed pregnancy duration, birth weight, and each z-score was assessed by multivariable linear regression models. Results were given with and without controlling for the difference between gestational age according to LMP and gestational age according to the ultrasound dating using HC. The ultrasound dating was based on charts constructed on the same population [12]. Values of pregnancy duration were derived by back transformation. We used the statistical package SPSS (Statistical Package for the Social Sciences; SPSS, Inc, Chicago, IL) and the SAS Software (SAS Institute, Cary, NC) for the analysis.

## Results

**Figure 1:**
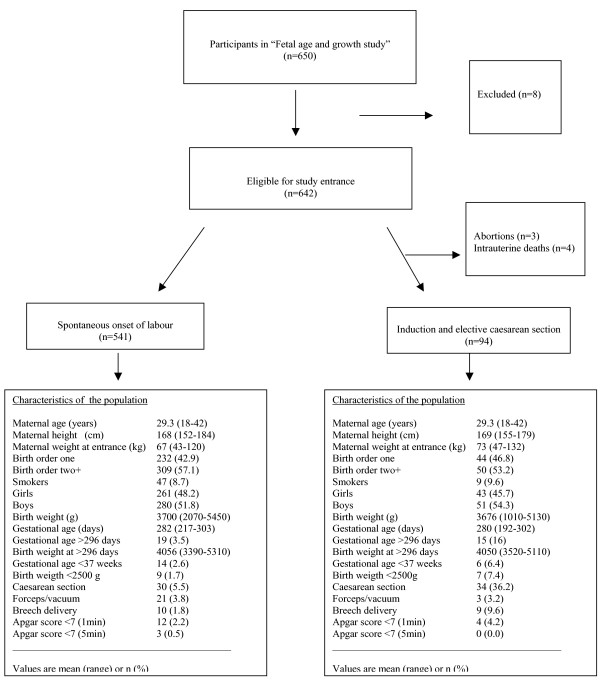
Flow chart, characteristics and outcome of the study population.

A flow chart and characteristics of the study population are presented in Figure 1.

### Duration of pregnancy

Small fetuses (measured by HC, AC or FL) had longer pregnancies than large fetuses (Table 1) (*p *< 0.0001). Z-scores of -2.5 for HC, AC and FL at 10–24 weeks of gestation were associated with pregnancy durations of 289.6, 289, and 288.3 days, respectively; corresponding values for z-scores of +2.5 were 276.1, 276.9, and 277.9 days. This corresponds to differences in pregnancy duration of 10–14 days for the extreme groups.

**Table 1:** Pregnancy duration according to fetal size at 10–24 weeks of gestation.

	HC		AC		FL	
			
z-score	Pregnancy duration (days)	95% CI	Pregnancy duration (days)	95% CI	Pregnancy duration (days)	95% CI
	Unadjusted results					
	
-2.5	289.6	288.0, 291.1	289.0	287.4, 290.6	288.3	286.6, 289.9
-2.0	288.5	287.1, 289.8	288.0	286.6, 289.4	287.4	286.0, 288.8
-1.5	287.3	286.2, 288.4	287.0	285.8, 288.1	286.5	285.3, 287.6
-1.0	286.1	285.2, 287.0	285.9	284.9, 286.8	285.6	284.6, 286.5
-0.5	284.9	284.1, 285.6	284.7	284.0, 285.5	284.6	283.8, 285.3
0.0	283.6	282.8, 284.2	283.5	282.8, 284.2	283.6	282.8, 284.3
0.5	282.2	281.4, 283.0	282.3	281.5, 283.1	282.5	281.7, 283.3
1.0	280.8	279.7, 281.8	281.1	280.0, 282.1	281.4	280.3, 282.5
1.5	279.3	277.8, 280.7	279.7	278.3, 281.1	280.3	278.9, 281.7
2.0	277.7	275.8, 279.5	278.3	276.4, 280.1	279.1	277.3, 280.9
2.5	276.1	273.6, 278.4	276.9	274.4, 279.2	277.9	275.6, 280.1
P-value†	<0.0001		<0.0001		<0.0001	

	Adjusted for the difference between LMP- and HC-based gestational ages.					
	
-2.5	286.9	282.9, 290.4	286.6	284.5, 288.6	286.0	284.1, 287.8
-2.0	286.3	283.0, 289.2	286.0	284.2, 287.7	285.5	283.9, 287.1
-1.5	285.6	283.1, 287.9	285.4	284.0, 286.8	285.1	283.8, 286.3
-1.0	284.9	283.2, 286.6	284.8	283.7, 285.8	284.6	283.5, 285.5
-0.5	284.2	283.2, 285.3	284.2	283.4, 285.0	284.1	283.3, 284.8
0.0	283.5	282.8, 284.2	283.5	282.8, 284.2	283.5	282.8, 284.2
0.5	282.8	281.7, 283.9	282.9	282.0, 283.7	283.0	282.2, 283.8
1.0	282.1	280.2, 283.9	282.2	281.1, 283.4	282.5	281.4, 283.5
1.5	281.3	278.5, 284.0	281.6	280.0, 283.1	282.0	280.5, 283.3
2.0	280.6	276.6, 284.0	280.9	278.8, 282.8	281.4	279.6, 283.2
2.5	279.8	274.7, 284.1	280.2	277.5, 282.6	280.9	278.6, 283.0
P-value†	0.089		0.004		0.008	

Pregnancy duration calculated from a certain and regular last menstrual period (LMP) in 541 pregnancies with spontaneous onset of labour are presented according to fetal size at 10–24 weeks of gestation, i.e. according to z-scores* of head circumference (HC), abdominal circumference (AC), and femur length (FL) in multivariable linear regression** (upper panel). Values for pregnancy duration when adjusted for the difference between LMP- and HC-based gestational ages are shown in the lower panel.

* Z-scores according to gestational age specific means and standard deviations of each biometric measure.

** To normalize the data pregnancy duration used in the regression model are transformed to the power of 10.

† Test for the association between z-score of each biometric measure and pregnancy duration.

Adjusting for the difference between LMP and ultrasound dating (based on HC-measurement at 10–24 weeks) by including this information in the multivariate regression analysis, the effect of fetal size on pregnancy duration was reduced by around half (Table 1), but remained significant for AC (286.6 vs. 280.2 days for z-score -2.5 vs. +2.5) and FL (286 vs. 280.9 days). Table 1 shows that, adjusting for the difference between LMP and ultrasound dating, the relations between early pregnancy fetal size and gestation duration were graded across the entire range of size, and did not depend on extremely small or large fetuses. Fetal gender, maternal weight, height, parity and smoking habits had no significant effect on pregnancy duration.

### Birth weight

Biometric size at 10–24 weeks of gestation was positively related to birth weight. We found that small HC, AC and FL (z-score -2) were associated with lower birth weight (3493, 3485 and 3656 gram, respectively) compared to those with large biometry (z-score +2) (3905, 3918 and 3787 gram, respectively) (*p *< 0.0001).

## Discussion

We have demonstrated a graded relation between fetal size at 10–24 weeks of gestation and pregnancy duration. Our data showed that fetuses with a second trimester size smaller than expected for their duration of gestation tended to have longer pregnancies compared to large fetuses (12 days between z-score -2.5 and +2.5 for AC) (Table 1). The slow growth of the small fetus is combined with an extended pregnancy duration that does not fully compensate for slow growth as shown by their lower birth weight. When our analysis adjusted for the difference between LMP and ultrasound assigned gestational age (using HC at 10–24 weeks), the effect of fetal size on pregnancy duration was reduced to a smaller but still significant difference; for AC the adjusted difference between z-score -2.5 and +2.5 was 6.4 days (Table 1). Such an adjustment may represent an over-correction of the results reducing biological variation in size and growth.

Adjusting for the difference between LMP- and ultrasound-based (HC) gestational age could possibly represent a confounder, but this is not obvious and in fact the effect of fetal size on pregnancy length is even stronger when not controlling for fetal head size in the second trimester. Since clinicians tend to use ultrasound to adjust fetal age, there is a risk of bias when fetuses with larger heads are assigned to a more advanced gestational age and possibly more inductions in the post-term period. Conversely, with the continuous focus that clinicians have on intra-uterine growth-restriction, it is more likely that the normally growing small fetus is at increased risk of induction and caesarean section. When including also such pregnancies in the analysis, an effect of fetal size on pregnancy length was still present (results not shown).

Several investigators have reported that fetuses smaller than expected in the second trimester have an increased risk of adverse obstetrical outcomes such as low birthweight [16] and premature birth [16,17]. These studies were population based and included both normal and growth restricted fetuses. A recent study of pregnancies following in vitro fertilisation demonstrated an increased risk for small for gestational age infants and premature birth (birth < 37 weeks of gestation), and a shorter duration of pregnancy when crown-rump length was less than expected at an early ultrasound scan [18]; however, the study did not take account of iatrogenic reduction of pregnancy duration by caesarean section or induction of labour. In contrast to the mentioned studies, the present study was based on a healthy population of low-risk women who spontaneously went into labour and the overall obstetric outcome was good, with a low incidence of low birth weight and premature births (Table 1). Thus, smaller fetuses in the present study can be regarded to be within normal biological variation, not growth restricted, and interestingly they had longer, not shorter duration of pregnancies.

Our results are supported by a recent study of the heritable component of duration of pregnancy [19]. In that study, the father and mother's own gestational age at birth were associated with the offspring's gestational age, and fathers with higher birth weights had larger offspring with shorter gestational length, which is in line with our results. The results of all the studies mentioned above fit with a U-shaped relationship between fetal growth and duration of pregnancy; rapidly growing fetuses tend to have shorter duration, slower growing fetuses a longer duration of pregnancy while pathologically slow growth have increased rates of birth before 37 weeks of gestation.

Six factors may influence our results: uncertain LMP [4], variation in ovulation and implantation [6,20-22], early growth restriction [16], random error of the ultrasound measurement [23] and biological variation in fetal size. In the present study the participants knew the exact date of their LMP and had a history of regular menstrual periods (28 ± 4 days) for at least three months before pregnancy. Extended duration of pregnancy could possibly compensate for underestimated age assessment due to delayed ovulation and implantation, but the present study showed that despite the extended duration of their pregnancies, fetuses found to be small in the second trimester also had lower birth weight.

The error of ultrasound measurement of the fetal head can be reduced by repeated measurements. We took the average of three measurements, as a result of which the error, counted in gestational days, is small (95% CI -1.5;1.5) [23].

According to the study protocol eight participants were excluded due to a discrepancy of more than 14 days between LMP and ultrasound dating. The choice of 14 days as cut off was based on reports that there is an increased risk of growth-restriction and adverse outcome in such pregnancies [24-26] and we aimed to study a low-risk population.

Wilcox et al. found that "the fertile window" starts ~5 days before ovulation and includes the day of ovulation with the highest probability of conception on the last day before ovulation [6]. Although this "fertility window" may occur within a wide range of a regular cycle, the follicular phase in natural cycles is fairly constant, with an SD of 3 days for most fertile women [22], suggesting that part of the variation (possibly half, i.e. ~6 days) that we see in pregnancy duration is attributable to ovulation variation, the rest being biological variation linking growth to pregnancy duration.

Discrepancies between gestational age assessed by a regular LMP and ultrasound may also be due to delayed implantation. There are data suggesting that later implantation is associated with an increased risk of early pregnancy loss [20], and that a large LMP-ultrasound discrepancy is associated with growth restriction and premature labour [24-26], possibly due to late implantation [20]. We believe these results represent extreme conditions that lead to abnormal pregnancies. The present results suggest that within physiological ranges fetal growth may be slower and then associated with an extended pregnancy, a phenomenon that is also known from animal studies [27]. Nutritionally restricted pregnancies resulted in slower growth and longer pregnancies.

The ultrasound method is better than certain menstrual history in predicting the date of spontaneous delivery [9,10], but ultrasound dating disregards biological variation in growth and pregnancy length. For fetuses smaller than the mean, ultrasound shifts this group to an artificially lower gestational age, and vice versa for fetuses larger than the mean. For example, differences of 2.5 days between male and female with regard to gestational age assessment based on ultrasound, have been reported [12]. This systematic shift leads to an artificially higher number of births defined as post-term among boys compared to girls [11,28], with a correspondingly higher rate of induction of labour in pregnancies with male fetuses. We believe that a similar error occurs when using ultrasound in normal pregnancies with certain information of a regular LMP, the consequence being an underestimation of gestational age and pregnancy duration in those with smaller head size than the mean, and vice versa for fetuses above the mean.

## Conclusion

To accommodate biological variation in fetal growth, customized [29] and conditional [30] models have been developed to individualize growth assessment. We believe that by accepting a greater biological variation of pregnancy duration than is imposed by the current ultrasound dating method, our clinical assessments may be founded on sounder biological principles.

## Competing interests

The authors declare that they have no competing interests.

## Authors' contributions

SLJ: contributed to conception and design of the study, acquisition of data, interpretation of data, drafting the manuscript, and approved the final version. TW: contributed with statistical analysis, interpretation of data and revision of the manuscript, and approved the final version. SR: contributed to conception and design of the study, analysis and interpretation of data, revision of the manuscript, and approved the final version. MAH, KG: contributed in interpretation of the data, revision of the manuscript and have given their final approval of this version to be published. TK: contributed to conception and design of the study, interpretation of data, helped to draft the manuscript, and approved the final version.

## Pre-publication history

The pre-publication history for this paper can be accessed here:


